# Astaxanthin attenuates pulmonary fibrosis through lncITPF and mitochondria‐mediated signal pathways

**DOI:** 10.1111/jcmm.15477

**Published:** 2020-08-19

**Authors:** Hongbin Chen, Jing Wang, Rongrong Li, Changjun Lv, Pan Xu, Youlei Wang, Xiaodong Song, Jinjin Zhang

**Affiliations:** ^1^ Department of Cellular and Genetic Medicine School of Pharmaceutical Sciences Binzhou Medical University Yantai China; ^2^ Department of Respiratory Medicine Affiliated Hospital to Binzhou Medical University Binzhou Medical University Binzhou China

**Keywords:** astaxanthin, lncRNA, mitochondrial signal pathway, pulmonary fibrosis

## Abstract

Pulmonary fibrosis is a chronic interstitial lung disease characterized by pulmonary epithelial injury, fibroblast activation, extracellular matrix deposition, and tissue structure destruction. However, an effective drug treatment remains unavailable. Therefore, studying the mechanism of pulmonary fibrogenesis and finding effective drugs have become important problems in the field of respiratory diseases. Pulmonary fibrosis is typically characterized by activated fibroblast proliferation and migration. Hence, abnormality in activated fibroblast proliferation and migration is a major concern for treating pulmonary fibrosis. Long noncoding RNA (lncRNA) is an enigmatic subclass of ncRNA that regulates various fundamental biological processes and participates in disease occurrence and development. However, studies on lncRNA as the therapeutic target of drug action are rarely reported. Our group first identified differentially expressed lncRNAs and revealed that lncITPF is a highly upregulated lncRNA in lung fibrosis. In particular, lncITPF is detected in the blood of patients with idiopathic pulmonary fibrosis. Clinical analysis shows that lncITPF is positively correlated with the degree of fibrosis. The receiver operating characteristic (ROC) curve indicates that the specificity and sensitivity values are 95.0 and 64.3, respectively. The area under the ROC curve is 0.804, indicating that lncITPF can be a diagnostic biomarker for IPF. However, whether lncITPF is effective as a therapeutic target of drug action against pulmonary fibrosis remains unclear. In this study, lncITPF acting as the therapeutic target of astaxanthin was explored in depth. The findings elucidated that astaxanthin blocks the activated fibroblast proliferation and migration through lncITPF and mitochondria‐mediated signal pathways to alleviate pulmonary fibrogenesis.

## 1 INTRODUCTION

Pulmonary fibrosis is a chronic interstitial lung disease characterized by pulmonary epithelial injury, fibroblast activation, extracellular matrix deposition and tissue structure destruction.^1^ However, an effective drug treatment remains unavailable.^2^ Therefore, studying the mechanism of pulmonary fibrogenesis and finding effective drugs have become important problems in the field of respiratory diseases. Pulmonary fibrosis is typically characterized by activated fibroblast proliferation and migration.^3^ Hence, abnormality in activated fibroblast proliferation and migration is a major concern for treating pulmonary fibrosis.

Long noncoding RNA (lncRNA) is an enigmatic subclass of ncRNA that regulates various fundamental biological processes and participates in disease occurrence and development.^4^, ^5^ However, studies on lncRNA as the therapeutic target of drug action are rarely reported. Our group first identified differentially expressed lncRNAs and revealed that lncITPF is a highly up‐regulated lncRNA in lung fibrosis.^6^, ^7^ In particular, lncITPF is detected in the blood of patients with idiopathic pulmonary fibrosis (IPF). Clinical analysis shows that lncITPF is positively correlated with the degree of fibrosis.^7^ The receiver operating characteristic (ROC) curve indicates that the specificity and sensitivity values are 95.0 and 64.3, respectively. The area under the ROC curve is 0.804,^7^ indicating that lncITPF can be a diagnostic biomarker for IPF. However, whether lncITPF is effective as a therapeutic target of drug action against pulmonary fibrosis remains unclear. In this study, lncITPF acting as the therapeutic target of astaxanthin was explored in depth. The findings elucidated that astaxanthin blocks the activated fibroblast proliferation and migration through lncITPF and mitochondria‐mediated signal pathways to alleviate pulmonary fibrogenesis.

## 2 MATERIALS AND METHODS

### 2.1 Animal model and ethics statement

Animal experiments were performed in accordance with the regulations of the Ethics Committee of Animal Experiments of Binzhou Medical University. The mice were divided into three groups, namely sham, bleomycin (BLM)‐treated and BLM + astaxanthin groups. The sham group only received saline at a volume equal to that of the treatments in other groups. The BLM model was administered with 5 mg/kg BLM dissolved in saline via single intratracheal instillation under anaesthesia. On day 3, 3 mg/kg astaxanthin was orally administered every day. On day 28, all of the mice were sacrificed. Haematoxylin and eosin (HE) and Masson's trichrome staining, pulmonary function analysis and Western blot were conducted in accordance with the previous study.^7^

### 2.2 Cell culture and treatment

Human foetal lung fibroblast MRC‐5 cells were maintained in Dulbecco's modified Eagle's medium containing 10% newborn calf serum, 100 U/mL penicillin, and 100 µg/mL streptomycin at 37°C under a humidified atmosphere of 5% CO_2_ and 95% air. The cells were first administered with 5 ng/mL transforming growth factor beta 1 (TGF‐β1) for 72 h and then cotreated with 24 µg/mL astaxanthin at different times in accordance with the experimental requirements. Real‐time cellular proliferation and migration were performed in accordance with the previous study.^7^

### 2.3 Wound healing assay

MRC‐5 cells (5 × 10^5^/mL) were seeded in a 96‐well plate and incubated in an incubator. After overnight incubation, the cells were wounded with cell scratcher, washed with PBS, replaced with complete medium culture, and placed in IncuCyte S3 (Essen BioScience) for real‐time dynamic observation. Images were taken on IncuCyte S3 software.

### 2.4 Analysis of astaxanthin autofluorescence

Astaxanthin was dissolved in methanol and configured with different concentrations of 10^−7^, 10^−6^, 10^−5^, 10^−4^ and 10^−3^ mol/L. Each sample was measured from 200 to 700 nm using a fluorescence spectrophotometer (Thermo Fisher Scientific Inc). Each test wavelength of stimulated fluorescence was increased by 2 nm.

### 2.5 Chromatin immunoprecipitation (ChIP)‐quantitative polymerase chain reaction (qPCR)

The cell samples treated with TGF‐β1 and cotreated with astaxanthin were divided into two groups. Anti‐Smad2/3 was purchased from Abcam Technology, and normal rabbit immunoglobulin G (IgG) was purchased from Cell Signaling Technology. The treated cells were lysed, collected and diluted using a ChIP dilution buffer. A total of 5 µL anti‐Smad2/3 and 3 µg IgG were added. After incubation overnight at 4°C, 40 µL protein G bead was added into the CHIP reaction system and sequentially washed in turn with low salt immune complex wash, high salt immune complex wash, LiCl salt immune complex wash and TE buffers. ChIP and input samples were simultaneously decross‐linked and tested through qRT‐PCR. The PCR conditions were as follows: initial denaturation at 95°C for 3 minutes, followed by 48 cycles of 95°C for 10 seconds, 60°C for 10 seconds and 72°C for 20 seconds.

### 2.6 Single‐molecule RNA fluorescence in situ hybridization (FISH)

LncITPF FISH probe was synthesized by Ribo Biotechnology. FISH was performed with a FISH kit in accordance with the manufacturer's protocol (Ribo Biotechnology). The cells were hybridized with 20 mmol/L using Cy3‐labelled RNA of lncITPF or U6/18S FISH probe mix in a moist chamber at 37°C overnight. The images were observed with a confocal microscope and analysed on fluorescence microscope software (Leica).

### 2.7 Statistical evaluation

Statistical analyses were performed using SPSS version 19.0 software. Data are presented as the mean ± standard deviation (SD) of at least three independent experiments. Statistical significance was considered at *P* < .05.

## 3 RESULTS AND DISCUSSIONS

### 3.1 LncITPF‐mediated antifibrotic mechanism of astaxanthin

**FIGURE 1:**
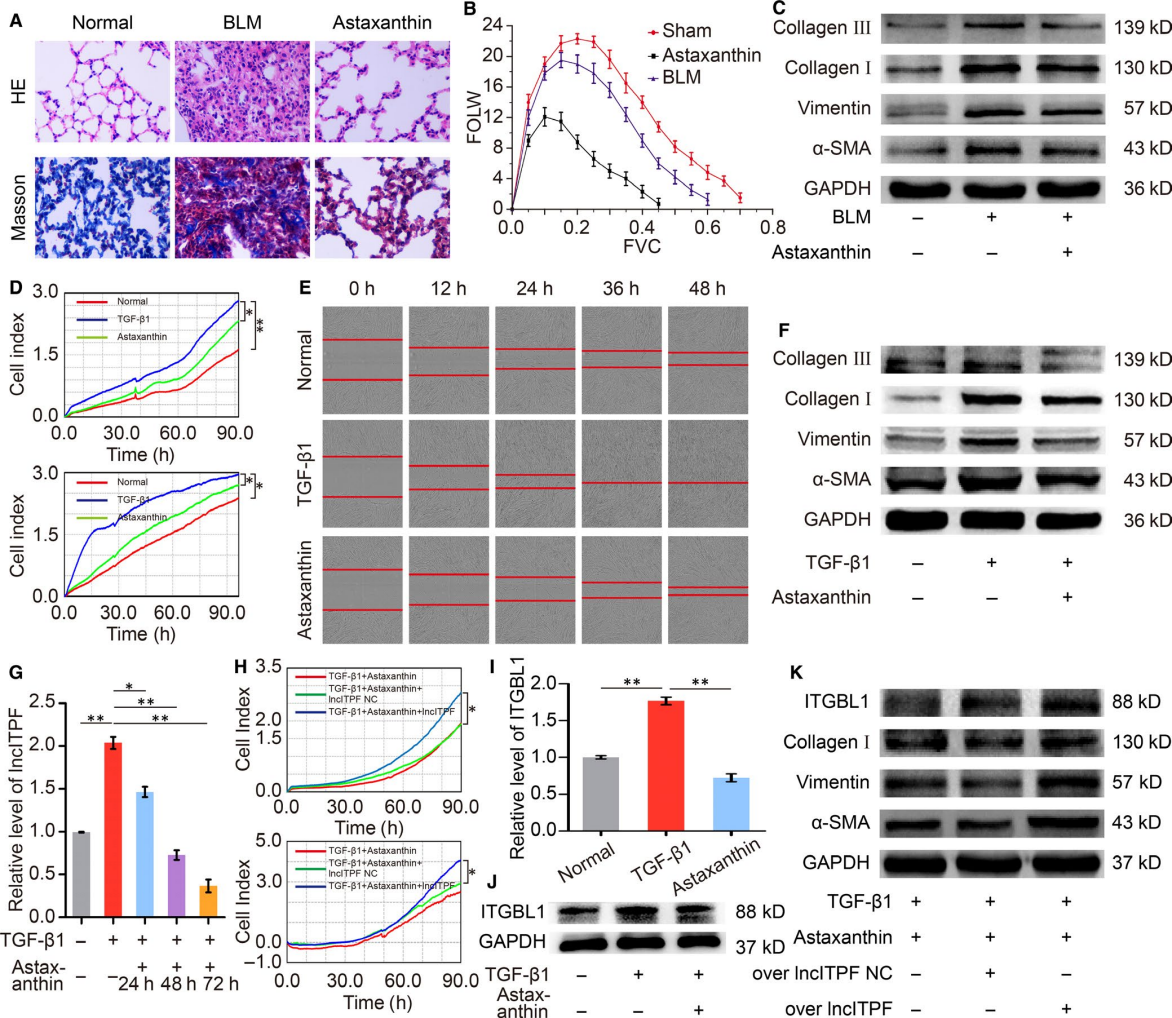
Inhibition of astaxanthin on activated fibroblast proliferation and migration through the lncITPF‐mediated signal pathway. A, HE and Masson's staining showed that astaxanthin improved the alveolar structure of mice, such as spacious alveolar space and thin alveolar wall, and reduced collagen fibres in the interstitial lung tissues of mice. B, Buxco PFT analysis system revealed that astaxanthin enhanced the pulmonary function of mice compared with those in the bleomycin group. C, Astaxanthin inhibited fibrotic markers α‐SMA, vimentin, collagen I and collagen III expression levels in mice. D, MRC‐5 cells were first administered with 5 ng/mL TGF‐β1 for 72 h and then treated with 24 µg/mL astaxanthin for 48 h. The cell samples were observed for 90 h using an RTCA DPlus instrument. The curves of proliferation/migration were automatically recorded. Real‐time cell analysis showed that astaxanthin strongly inhibited TGF‐β1‐treated cell proliferation/migration compared with the TGF‐β1‐treated group. E, Images automatically monitored by an IncuCyte S3 instrument confirmed that astaxanthin repressed the migration of TGF‐β1‐treated MRC‐5 cells at different time points. F, Astaxanthin significantly reduced the expression levels of α‐SMA, vimentin, collagen I and collagen III in MRC‐5 cells. G, Astaxanthin reduced the lncITPF expression in MRC‐5 cells through qRT‐PCR. (H) The rescue experiment showed that the overexpressed lncITPF reversed the astaxanthin effect on activated fibroblast proliferation and migration in MRC‐5 cells. I and J, Astaxanthin inhibited the ITGBL1 expression at the mRNA and protein levels through qRT‐PCR and Western blot. K, A rescue experiment was conducted to elucidate the antipulmonary fibrotic effect of astaxanthin depending on lncITPF. The results demonstrated that astaxanthin attenuated the high collagen, vimentin, α‐SMA and ITGBL1 expression levels caused by TGF‐β1. However, highly up‐regulated lncITPF reversed the antifibrotic effect of astaxanthin by increasing these protein expression levels. Each bar represents mean ± SD, n = 6, *P < 0.05, **P < 0.01

Astaxanthin is a xanthophyll carotenoid that can be transported into the brain through the blood‐brain barrier.^8^ In addition to its antioxidant activity, astaxanthin exhibits anti‐inflammatory and antitumour properties.^9^, ^10^, ^11^ However, limited studies report its antipulmonary fibrotic effect. The present study found that astaxanthin attenuated pulmonary fibrosis by blocking the activated fibroblast proliferation and migration, and lncITPF contributed to the antifibrotic mechanism (Figure 1A–H).

**FIGURE 2:**
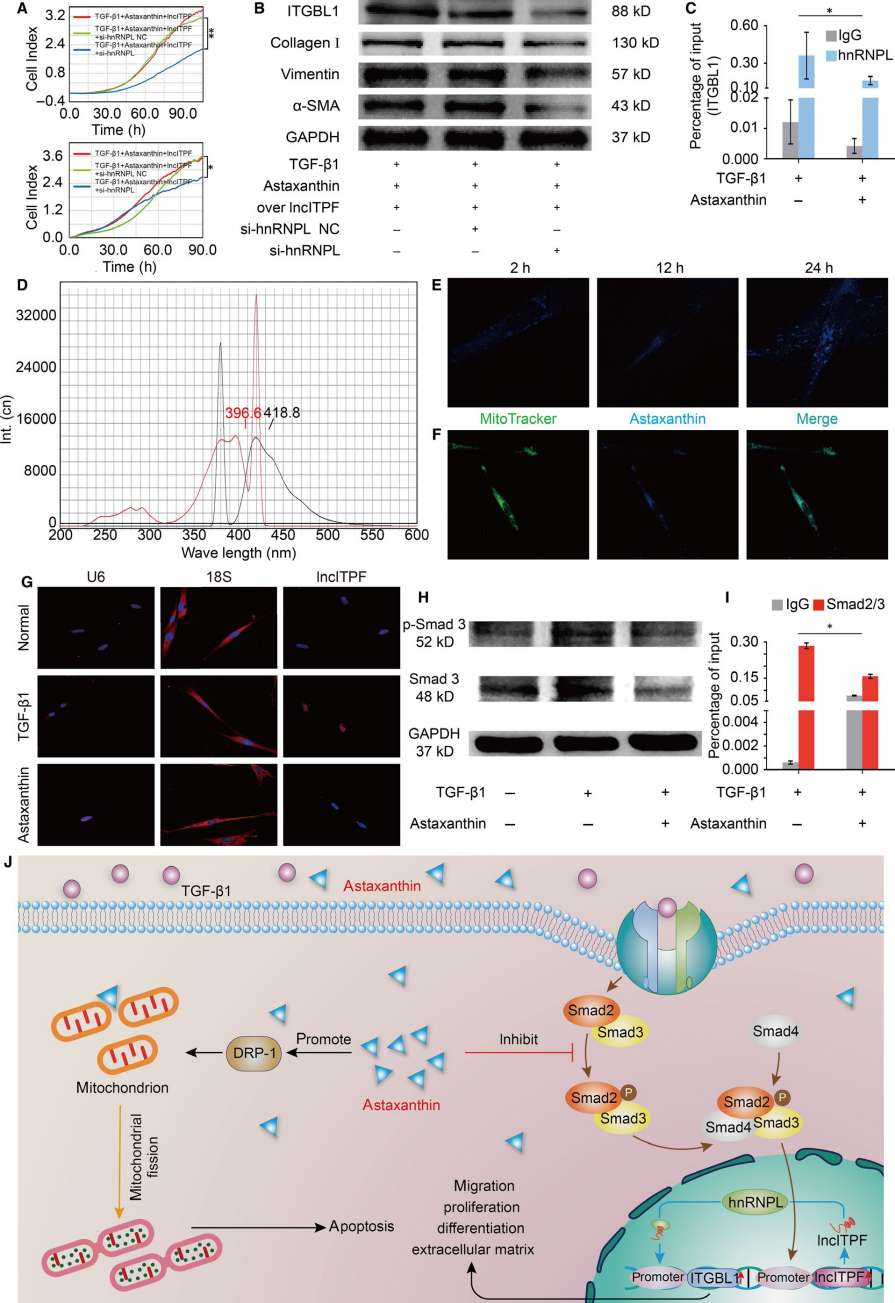
Antifibrotic pathway of astaxanthin in activated fibroblast. A, The rescue experiment showed that hnRNP L knockdown reversed the overexpressed lncITPF effect on activated fibroblast proliferation and migration in MRC‐5 cells. B, The rescue experiment showed that the overexpressed lncITPF reversed the astaxanthin effect to enhance the high collagen, vimentin, α‐SMA and ITGBL1 expression levels. HnRNP L knockdown reversed the effect of overexpressed lncITPF to decrease the collagen, vimentin, α‐SMA and ITGBL1 expression levels. The data indicated that the antifibrotic effect of astaxanthin was dependent on lncITPF − hnRNP L. C, CHIP‐qPCR assessed the binding of ITGBL1 on the promoter region of hnRNP L under the astaxanthin treatment. The data showed that astaxanthin induced a considerable decrease in the binding amount of ITGBL1 with the hnRNP L promoter. D, Astaxanthin autofluorescence showed that its excitation and emission wavelengths were 396.6 and 418.8 nm using a fluorescence spectrophotometer. E, Astaxanthin autofluorescence elucidated that astaxanthin penetrated the cells with time under a confocal microscope. F, The double‐labelling immunofluorescence experiments verified that a small amount of astaxanthin was localized in the mitochondria. MitoTracker is a specific probe used to detect mitochondria. G, RNA FISH showed that lncITPF was mainly located in the nucleus. Astaxanthin did not cause its translocation from the nucleus to the cytoplasm but induced a rapid decrease in the lncITPF level in the nucleus. H, P‐Smad3 decreased after the cells were treated with astaxanthin compared with the TGF‐β1‐stimulated cells. I, The CHIP‐qPCR experiment showed the binding of Smad2/3 with the promoter region of lncITPF. Astaxanthin reduced the binding of Smad2/3 in the lncITPF promoter region. J, The antipulmonary fibrotic pathways of astaxanthin are as follows: astaxanthin blocks the activated fibroblast proliferation and migration through the lncITPF‐mediated signal pathway. Astaxanthin promotes the activated fibroblast apoptosis through the mitochondrial fission signal pathway. Each bar represents mean ± SD, n = 6, *P < 0.05, **P < 0.01

We elucidated the lncITPF‐mediated mechanism of astaxanthin. LncRNA generally exerts its function through its host gene or/and binding protein.^12^, ^13^ Our previous work revealed that lncITPF promotes pulmonary fibrosis by binding heterogeneous nuclear ribonucleoprotein (hnRNP) L to control its host gene integrin b‐like 1 (ITGBL1). The present study demonstrated that astaxanthin intensively repressed ITGBL1. A rescue experiment identified that astaxanthin attenuated the high collagen, vimentin, α‐SMA and ITGBL1 expression levels caused by TGF‐β1. However, highly up‐regulated lncITPF reversed the antifibrotic effect of astaxanthin (Figure 1I–K), indicating that this effect depended on lncITPF. Another gain/loss‐of‐function and rescue experiments were performed to elucidate the antipulmonary fibrotic effect of astaxanthin depending on lncITPF‐hnRNP L. HnRNP L knockdown enhanced the antifibrotic effect of astaxanthin by strongly repressing the activated fibroblast proliferation and migration. Astaxanthin decreased the high collagen, vimentin, α‐SMA and ITGBL1 expression levels caused by TGF‐β1. Overexpressed lncITPF reversed the astaxanthin effect, and hnRNP L knockdown reversed the effect of overexpressed lncITPF (Figure 2A–B). These findings indicated the antifibrotic effect of astaxanthin depended on lncITPF − hnRNP L. CHIP‐qPCR exhibited that astaxanthin induced a considerable decrease in the binding amount of ITGBL1 with hnRNP L promoter (Figure 2C). All the above findings reflected that astaxanthin blocked the activated fibroblast proliferation and migration by lncITPF binding hnRNP L to target ITGBL1.

### 3.2 Antifibrotic pathway of astaxanthin in activated fibroblast

To elucidate the antifibrotic pathway of astaxanthin in the activated fibroblast, astaxanthin autofluorescence and colocalization experiments were performed to trace its distribution in cells. The results showed that astaxanthin penetrated the cytoplasm, and a small amount of astaxanthin was distributed in the mitochondria (Figure 2D–F). Our previous study identified that astaxanthin promotes activated fibroblast apoptosis by regulating dynamin‐related protein 1 (Drp‐1)‐mediated mitochondrial fission.^14^ A recent study suggested that the Drp‐1 localized in mitochondria is associated with mitochondrial division and apoptosis in ischaemic acute kidney injury.^15^ The colocalized phenomenon of astaxanthin and mitochondria validated our previous conclusion that astaxanthin partially attenuated pulmonary fibrosis by regulating Drp‐1‐mediated mitochondrial fission to promote the apoptosis of activated fibroblasts.

RNA FISH depicted that lncITPF was mainly located in the nucleus with or without astaxanthin treatment, thereby indicating that astaxanthin only induced a rapid decrease in lncITPF level in the nucleus and did not cause lncITPF translocation from the nucleus to the cytoplasm (Figure 2G). However, few empirical evidence showed that astaxanthin can enter the nucleus, and the regulation of lncITPF by astaxanthin remains unclear. Given that Smad3 functions as a transcriptional regulator of lncITPF,^7^ the effect of astaxanthin on Smad3 phosphorylation was determined. P‐Smad3 decreased after astaxanthin treatment, suggesting that the p‐Smad3 content in the nucleus decreased and astaxanthin blocked Smad3 translocation from the cytoplasm to the nucleus (Figure 2H). CHIP experiments were performed to investigate the binding of Smad3 with the promoter region of lncITPF under astaxanthin action. The results showed that the bound amount of Smad3 significantly reduced in the lncITPF promoter after astaxanthin treatment, indicating that astaxanthin alleviated lung fibrosis by blocking Smad3 nuclear translocation to control lncITPF.

This study suggested that astaxanthin alleviated fibrogenesis through lncITPF‐ and mitochondria‐mediated signal pathways (Figure 2J). The data on the mechanism of astaxanthin would provide insight into the therapeutic drug for pulmonary fibrosis.

## CONFLICT OF INTEREST

No conflicts of interest, financial or otherwise, are declared by the authors.

## AUTHOR CONTRIBUTIONS

HB Chen, J Wang and RR Li performed the experiments and analysed the data. P Xu and YL W contributed to animal experiments and data analysis. CJ Lv, JJ Zhang, and XD Song designed the experiments and wrote the paper.

## Data Availability

I confirm that the data in the manuscript are truly usable.
